# Postoperative reduction in thrombin generation induced by elevated levels of tissue factor pathway inhibitor in cardiac surgery: a prospective observational study

**DOI:** 10.1007/s00540-026-03657-4

**Published:** 2026-01-13

**Authors:** Akiko Kitajo, Nobuyo Umehara, Aya Takemoto, Yudai Yamamoto, Yoshiki Sento, Tomoyuki Fujita, Tokujiro Uchida

**Affiliations:** 1 Department of Anesthesiology, Graduate School of Medical and Dental Sciences, Institute of Science Tokyo, 1-5-45 Yushima, Bunkyo-Ku, Tokyo, 113-8510 Japan; 2 Department of Cardiovascular Surgery, Graduate School of Medical and Dental Sciences, Institute of Science Tokyo, 1-5-45 Yushima, Bunkyo-Ku, Tokyo, 113-8510 Japan

**Keywords:** Cardiac surgery, Thrombin generation, Tissue factor pathway inhibitor

## Abstract

**Purpose:**

Tissue factor pathway inhibitor (TFPI) is an intrinsic anticoagulant factor, and its plasma concentration is elevated by heparin administration. Because several hours are required to return to normal range after heparin reversal, we investigated the role of TFPI in the inhibition of thrombin generation (TG) in patients undergoing cardiac surgery.

**Methods:**

Blood samples were collected from adult patients undergoing cardiac surgery with cardiopulmonary bypass (CPB) before, at the end of, and 1 day after surgery. Plasma concentration of TFPI, peak height of the TG assay (peak TG), and whole blood coagulation time by dielectric blood coagulometry using a Russell’s viper venom cartridge system were evaluated. Nonparametric correlation was evaluated using Spearman’s method, and time-dependent change was analyzed using repeated measures analysis of variance.

**Results:**

The plasma concentration of TFPI was higher (54 [47–60] ng/mL vs. 18 [13–27] ng/mL; *P* < 0.001) and the peak TG value was lower (98.1 [48.9–148] nM vs. 268 [244–309]; *P* < 0.001) at the end of surgery than before surgery. Plasma TFPI concentration showed a positive correlation with whole blood coagulation time as measured by dielectric blood coagulometry (Rs = 0.643) and a negative correlation with peak TG (Rs =  − 0.624). Anti-TFPI antibody neutralized reduction in peak TG.

**Conclusions:**

In patients undergoing cardiac surgery using CPB, the increase in plasma TFPI concentration at the end of surgery causes a reduction in TG and impairment of whole blood coagulation via a mechanism that includes inhibition of factor Xa activity.

**Supplementary Information:**

The online version contains supplementary material available at https://doi.org/10.1007/s00540-026-03657-4.

## Introduction

Hemostasis can be difficult to achieve in patients undergoing cardiac surgery using cardiopulmonary bypass (CPB), even with sufficient protamine neutralization of heparin. Multiple factors might explain this, including hypothermia, platelet dysfunction, consumptive depletion of coagulation factors, dilutional coagulopathy, and the heparin rebound phenomenon [1].

Recent discussions have highlighted the reduction of thrombin generation (TG) during cardiac surgery involving CPB [2–4]. Impaired TG results in delayed clot formation, reduced hemostatic efficiency, and an increased risk of postoperative bleeding. The underlying mechanisms, however, remain to be fully elucidated. In clinical practice, this issue is addressed through replacement therapies aimed at restoring adequate hemostasis.

Tissue factor pathway inhibitor (TFPI) is a serine protease inhibitor which contains a Kunitz domain that exerts anticoagulant activity by binding to tissue factor–factor VII complex and factor X [5]. It is abundantly expressed on the surface of endothelial cells and increases in plasma concentration after heparin administration. Following protamine administration after CPB, the pre-CPB TFPI concentration is not restored again for several hours [6]. Therefore, we hypothesized that TFPI might play a role in inhibiting the coagulation cascade and reducing TG.

This study examined the change in plasma TFPI concentration over time and the role of TFPI in inhibition of TG in patients undergoing cardiac surgery. First, we evaluated whether postoperative prolongation of coagulation in whole blood samples treated with Russell’s viper venom could be detected as inhibition of factor Xa activity in a concentration-dependent manner with respect to TFPI. Second, we examined whether elevated TFPI concentration is associated with reduced TG, and whether neutralization of TFPI with anti-TFPI antibodies restores TG.

## Methods

We conducted a prospective observational study of patients undergoing elective major cardiac surgery using CPB at the Institute of Science Tokyo Hospital. Patients under the age of 20 years and those who underwent off-pump coronary bypass graft surgery were excluded. We also excluded patients whose blood sampling was incomplete. Institutional ethics committee approval was obtained (M2023-106 [August 24, 2023]). All participants provided written informed consent. The study was conducted in accordance with the principles of the Declaration of Helsinki.

### Blood sample collection and preparation

Samples were obtained at three time points by drawing 20 mL of blood from an arterial line inserted at induction of anesthesia: (1) before surgery (baseline), (2) at the end of surgery, and (3) on postoperative day (POD) 1. From each blood sample, a 2 mL aliquot was dispensed into an EDTA collection tube; the remaining 18 mL were dispensed into 3.2% sodium citrate collection tubes to perform dielectric blood coagulometry, TFPI concentration assessment, and coagulation parameter measurements including TG assay. The samples in the 3.2% sodium citrate collection tubes were centrifuged for 10 min (4 °C, 2000 rpm). Plasma was collected as platelet-poor plasma (PPP) samples.

### Complete blood count measurement

Complete blood count was measured using the automated MEK-1302 hematology analyzer (Nihon Koden Corp., Tokyo, Japan).

### Coagulation parameter measurements

The CS-1600 automatic coagulation test system (Sysmex Corporation, Kobe, Japan) was used to measure PT–INR, APTT, and fibrinogen concentration in PPP samples.

### TFPI concentration measurement

Plasma TFPI concentration was measured using the Human TFPI Enzyme-Linked Immunosorbent Assay Kit (RayBiotech, Peachtree Corners, GA, USA) and the iMark Microplate Absorbance Reader (Bio-Rad Laboratories, Hercules, CA, USA).

### Measurement of coagulation time after activation by Russell’s viper venom

We assessed coagulation time in whole blood samples after adding Russell’s viper venom, which directly activates factor X, to determine whether factor X-initiated coagulation is impaired. For this purpose, dielectric blood coagulometry was performed using a system developed for analysis of anticoagulation activity of factor Xa inhibitors [7]. Briefly, a dielectric blood coagulometry beta prototype (Sony Corporation, Tokyo, Japan) with a cartridge system specially designed to analyze anticoagulation activity in patients taking factor Xa inhibitors was used. Within the cartridge, Russell’s viper venom was added to calcium chloride to activate factor X, and coagulation time is prolonged by the substance inhibiting factor Xa in each sample. In each measurement, 200 μL of the whole blood sample was dispensed into the cartridge, and the change in dielectric permittivity was measured under a 1 MHz alternating current. The time from the sample loading to the peak of electric permittivity was defined as coagulation time. A clinical trial using this system demonstrated its usefulness in evaluating the anticoagulation effect induced by factor Xa inhibitors in patients undergoing elective surgery [7].

### TG assay

TG was assessed using the Calibrated Automated Thrombogram system (Thrombinoscope, Maastricht, The Netherlands) according to the manufacturer’s protocol. PPP samples were thawed at 37 °C prior to the assay. For each reaction, 80 µL of PPP was mixed with 20 µL of reaction reagent (PPP reagent; Thrombinoscope; final concentration: 5 pM tissue factor and 4 µM phospholipid) in polypropylene round-bottom microplates (Thermo Fisher Scientific, Tokyo, Japan). TG was initiated by the addition of 20 µL of FluCa-Kit (Thrombinoscope) and monitored by fluorescence.

Data were analyzed using Thrombinoscope software (Thrombinoscope). Peak TG was defined as the maximum value of thrombin concentration reached during the measurement. To evaluate the effects of TFPI and residual heparin on TG, the following four conditions were tested on the postoperative samples supplemented with 8.5 µL of the following treatment solutions: (1) buffered saline, (2) 1.44 µg of anti-human TFPI antibody (AF2974 R&D Systems, Inc., Minneapolis, MN, USA), (3) 0.65 U of heparinase I from *Flavobacterium heparinum* (H2519; Sigma-Aldrich, St. Louis, MO, USA), and (4) both anti-TFPI antibody (1.44 µg) and heparinase (0.65 U).

### Statistical analysis

Data are shown as medians with interquartile range. Nonparametric correlation was evaluated using Spearman’s method. The correlation between log-transformed peak TG value and TFPI was assessed using Pearson’s method. Changes in TFPI concentration, peak TG, PT–INR, APTT, and fibrinogen values over time were examined using repeated measures analysis of variance; post hoc comparisons were performed using the Bonferroni correction. The effect of TFPI-neutralizing antibody and heparinase on the linear regression equation between TFPI concentration and log-transformed peak TG value was examined using analysis of covariance. *P* < 0.05 was considered significant. Statistical analyses were performed using R version 4.3.1 (www.r-project.org), including libraries pROC, ez, and car.

## Results

### Patients

**Fig. 1:**
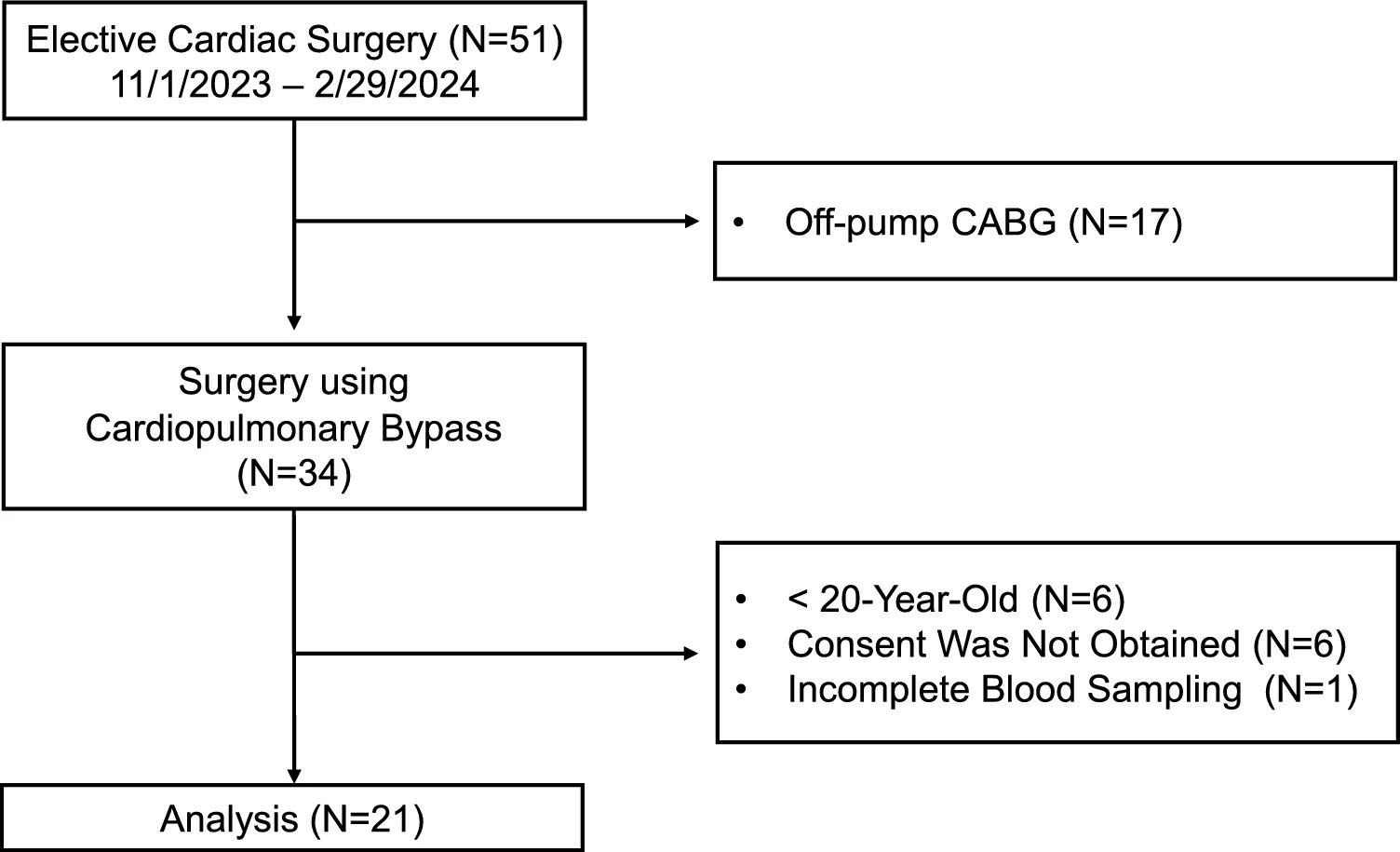
Study flowchart

Among 51 patients who underwent cardiac surgery between November 1, 2023, and February 29, 2024, CPB was used in 34 cases. Six of these patients were under 20 years of age, six did not provide consent, and blood sample collection was incomplete in one. Therefore, 21 patients were included in the study (Fig. 1). The characteristics of the patients and surgical procedures are shown in Table 1.

**Table 1:** Patient characteristics

Sex, male	9 (42.9)
Age (years)	61 [51–72]
Height (cm)	157 [153–165]
Body weight (kg)	57.3 [50.5–66.2]
Body mass index (kg/m^2^)	22.3 [21.3–26.1]
Antiplatelet	6 (28.6)
Anticoagulant	6 (28.6)
Preoperative heparinization	2 (9.5)
Platelets (10,000/μL)	19.1 [16.3–23.6]
Fibrinogen (mg/dL)	319 [258–410]
PT–INR	0.910 [0.830–0.940]
APTT (sec)	28.9 [26.5–33.5]
Surgical procedure	
Single valve replacement or valvuloplasty	10 (47.6)
Coronary artery bypass	1 (4.76)
Aortic surgery	2 (9.52)
Ventricular assisted device-associated	2 (9.52)
Others (combined procedures)	6 (28.6)
Bleeding (g)	1137 [462–2700]
Transfusion	
Red blood cells (mL)	0 [0–560]
Number of patients (%)	10 (47.6)
Fresh frozen plasma (mL)	480 [240–480]
Number of patients (%)	16 (76.2)
Platelet concentrate (U)	0 [0–20]
Number of patients (%)	9 (42.9)
Cardiopulmonary bypass blood (g)	430 [290–460]
Number of patients (%)	20 (95.2)
Intraoperative blood salvage (g)	380 [248–751]
Number of patients (%)	17 (81.0)
Cryoprecipitate (U)	0 [0–150]
Number of patients (%)	7 (33.3)
Surgical duration (minutes)	309 [258–464]
Duration of cardiopulmonary bypass (minutes)	153 [134–212]
Total heparin dose (10^3^U)	32.7 [27.2–42.6]

PT–INR, prothrombin time-international normalized ratio; APTT, activated partial thromboplastin time

Data shown are medians [interquartile range] or numbers (percentage)

Laboratory data at the three sampling points are summarized in Table 2. PT–INR at the end of surgery was prolonged compared with the time before surgery; however, the interquartile range remained within the normal range even at the end of surgery. On POD 1, PT–INR returned to the preoperative level. Both hematocrit and platelet count at the end of surgery decreased from preoperative levels, however, the difference was relatively small, and the interquartile ranges were above the trigger levels for transfusion, which was acceptable to finish surgery. The activated clotting time after protamine reversal was 120 [107–131] s, and APTT at the end of surgery was 31.5 [28.5–34.3] s (Table 2).

**Table 2:** Laboratory data during the perioperative period

	Baseline	End of surgery	POD 1
Hemoglobin (g/dL)	11.4 [10.4–12.2]	10.2 [9.7–10.9]	10.7 [10.1–11.3]
Platelets (10,000/μL)	15.6 [14.3–19.6]	11.7 [9.0–14.1]	10.6 [7.6–12.5]
Fibrinogen (mg/dL)	272.2 [235.4–313.8]	220.2 [189.3–247.6]	299.0 [247.2–321.8]
PT–INR	1.08 [1.03–1.15]	1.27 [1.19–1.37]	1.06 [1.03–1.10]
APTT (s)	31.6 [28.4–33.4]	31.5 [28.5–34.3]	25.8 [24.3–27.2]

PT–INR, prothrombin time–-international normalized ratio; APTT, activated partial thromboplastin time; POD 1, postoperative day 1

Data shown are medians [interquartile range] or numbers (percentage)

### Changes in plasma concentration of TFPI and its relationship with whole blood anti-factor Xa activity

**Fig. 2:**
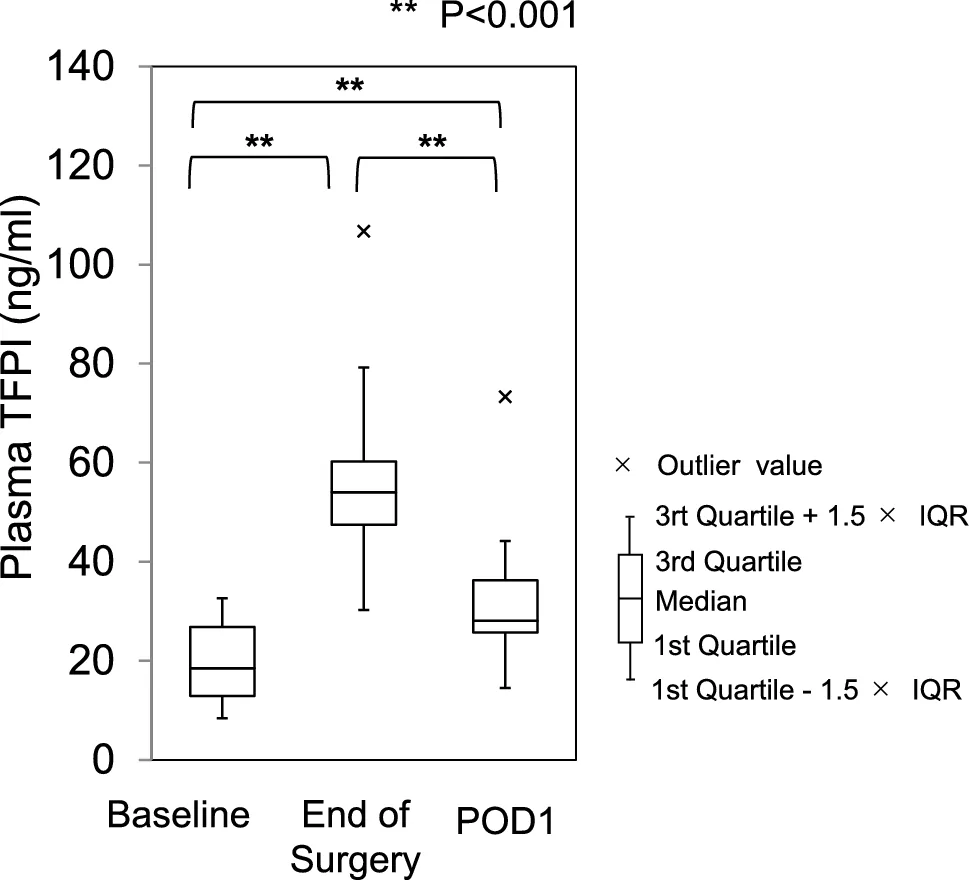
Perioperative change in plasma concentration of tissue factor pathway inhibitor. TFPI, tissue factor pathway inhibitor; TG, thrombin generation; POD, postoperative day; IQR, interquartile range

The plasma concentration of TFPI was significantly higher at the end of surgery than at baseline (54 [47–60] ng/mL vs. 18 [13–27] ng/mL; *P* < 0.001). Although it decreased on POD 1 compared with at the end of surgery (28 [26–36] ng/mL; *P* < 0.001), it remained significantly higher than the baseline level (*P* < 0.001; Fig. 2).

**Fig. 3:**
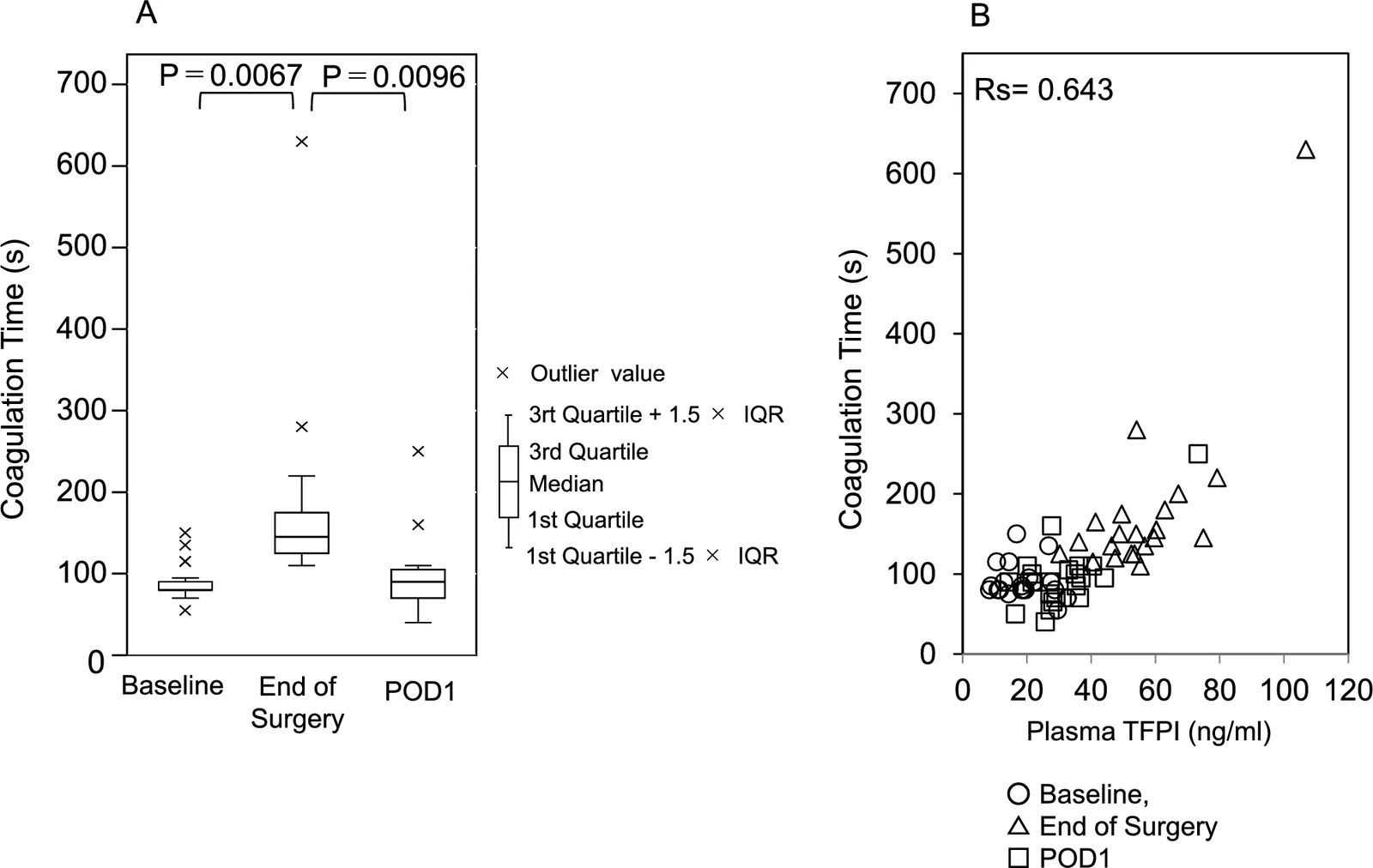
Perioperative change in whole blood coagulation time after activation by Russell’s viper venom and its relationship with plasma concentration of TFPI**. **(A) Perioperative change in coagulation times after activation by Russell’s viper venom measured by dielectric blood coagulometry at each blood sampling point. (B) Relationship between coagulation time measured by dielectric blood coagulometry and plasma TFPI concentration. A positive correlation was observed based on Spearman’s rank correlation test (Rs = 0.643). TFPI, tissue factor pathway inhibitor; TG, thrombin generation; POD, postoperative day; IQR, interquartile range

Figure 3 shows the distribution of plasma concentrations of TFPI and coagulation times measured by dielectric blood coagulometry at each blood sampling point. Similar to TFPI concentration, coagulation time was significantly longer at the end of surgery than at baseline (145 [125–175] s vs. 80 [80–90] s; *P* = 0.0067). It shortened on POD 1 compared with at the end of surgery (90 [70–105] s; *P* = 0.0096), and it returned to a level that did not differ significantly from the baseline value (*P* = 1.00). These two parameters were highly correlated (Rs = 0.643), indicating that Russell’s viper venom-induced coagulation was prolonged in proportion to TFPI concentration. While Russell’s viper venom directly activates factor X and triggers TG, at the end of surgery, these reactions were inhibited, and the degree of inhibition correlated with TFPI concentration.

### Reduction in TG in PPP after cardiac surgery depends on TFPI concentration

**Fig. 4:**
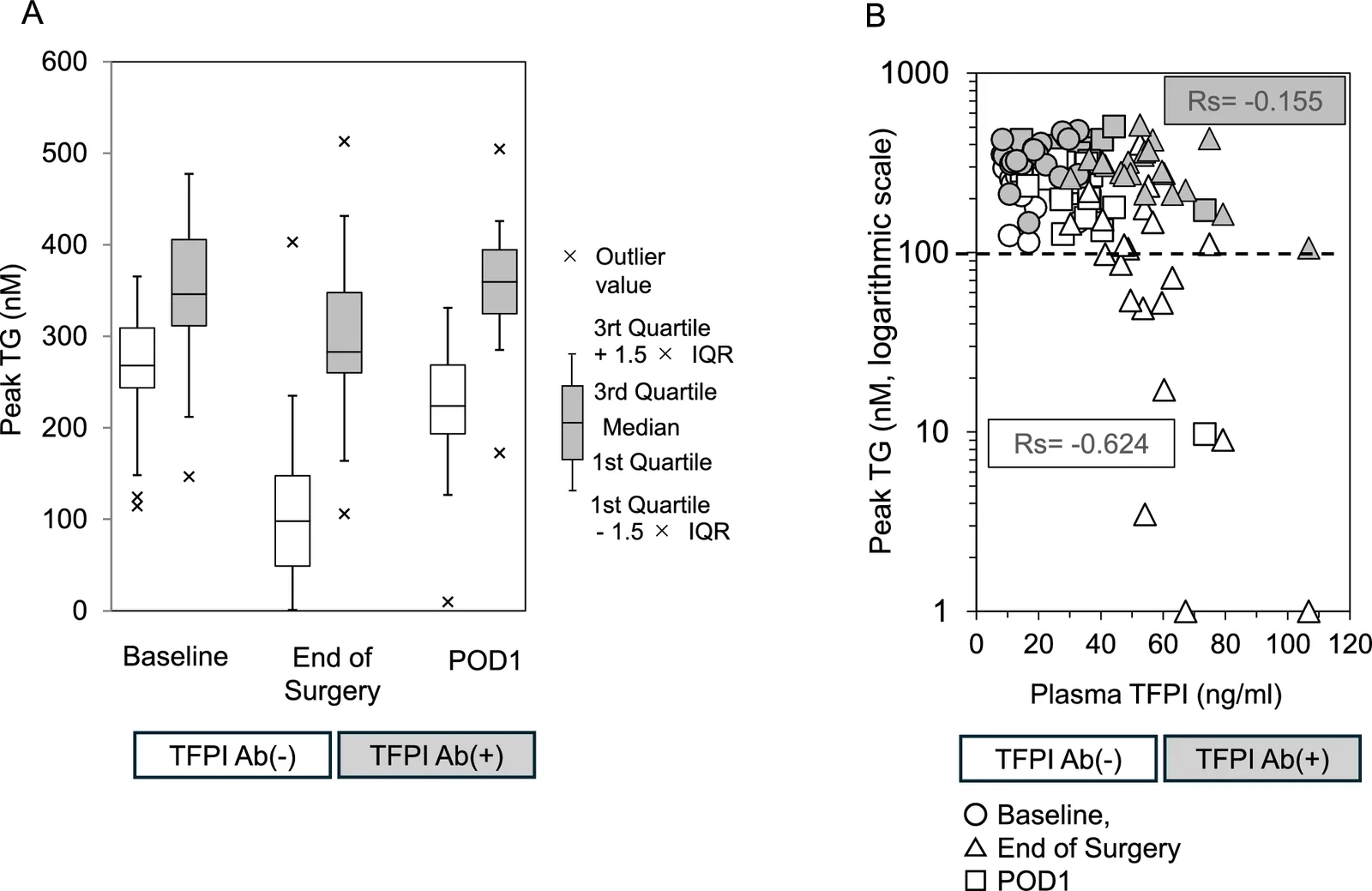
Perioperative change in peak thrombin generation (TG) and the effect of anti-tissue factor pathway inhibitor (TFPI) antibody. **A** Reduction of peak TG was observed at the end of surgery (white box-whisker plots). This reduction in peak TG value was ameliorated by neutralization using anti-TFPI antibody (gray box–whisker plots). **B** Negative correlation between peak TG value and plasma TFPI concentration at the three sampling points (white plots, Rs =  − 0.624). Reduction in peak TG was restored by anti-TFPI antibody treatment in each sample (gray plots; Rs =  − 0.155). TG, thrombin generation; TFPI, tissue factor pathway inhibitor; POD, postoperative day; IQR, interquartile range

In PPP, peak TG was significantly lower after surgery than at baseline (98.1 [48.9–148] nM vs. 268 [244–309] nM; *P* < 0.001). It increased on POD 1 compared with at the end of surgery (224 [193–268] nM; *P* = 0.0012), and it returned to a level that did not differ significantly from the baseline value (*P* = 0.125; white box–whisker plots in Fig. 4A).

Spearman’s ranked correlation test showed a significant negative correlation between peak TG value and plasma TFPI concentration (Rs =  − 0.624). Among the baseline samples, which were distributed below the maximum TFPI concentration of 33 ng/mL, none exhibited a peak TG value < 100 nM. In contrast, a peak TG value < 100 nM occurred in 11 of the 20 samples at the end of surgery with TFPI concentration ≥ 33 ng/mL (Fig. 4B).

To investigate the contribution of TFPI to reduction in TG, anti-TFPI antibody was added to each sample, which resulted in a significant increase in peak TG value at baseline (*P* < 0.001), at the end of surgery (*P* < 0.001), and on POD 1 (*P* < 0.001; gray box–whisker plots in Fig. 4A; gray plots in Fig. 4B). Repeated measures analysis of variance demonstrated that the reduction in peak TG observed at the end of surgery was abolished in the presence of anti-TFPI antibody, suggesting that TFPI suppresses TG (*P* < 0.001 for the effect of anti-TFPI antibody, *P* < 0.001 for the effect of sampling time, and *P* < 0.001 for the interaction between anti-TFPI antibody and sampling time).

**Fig. 5:**
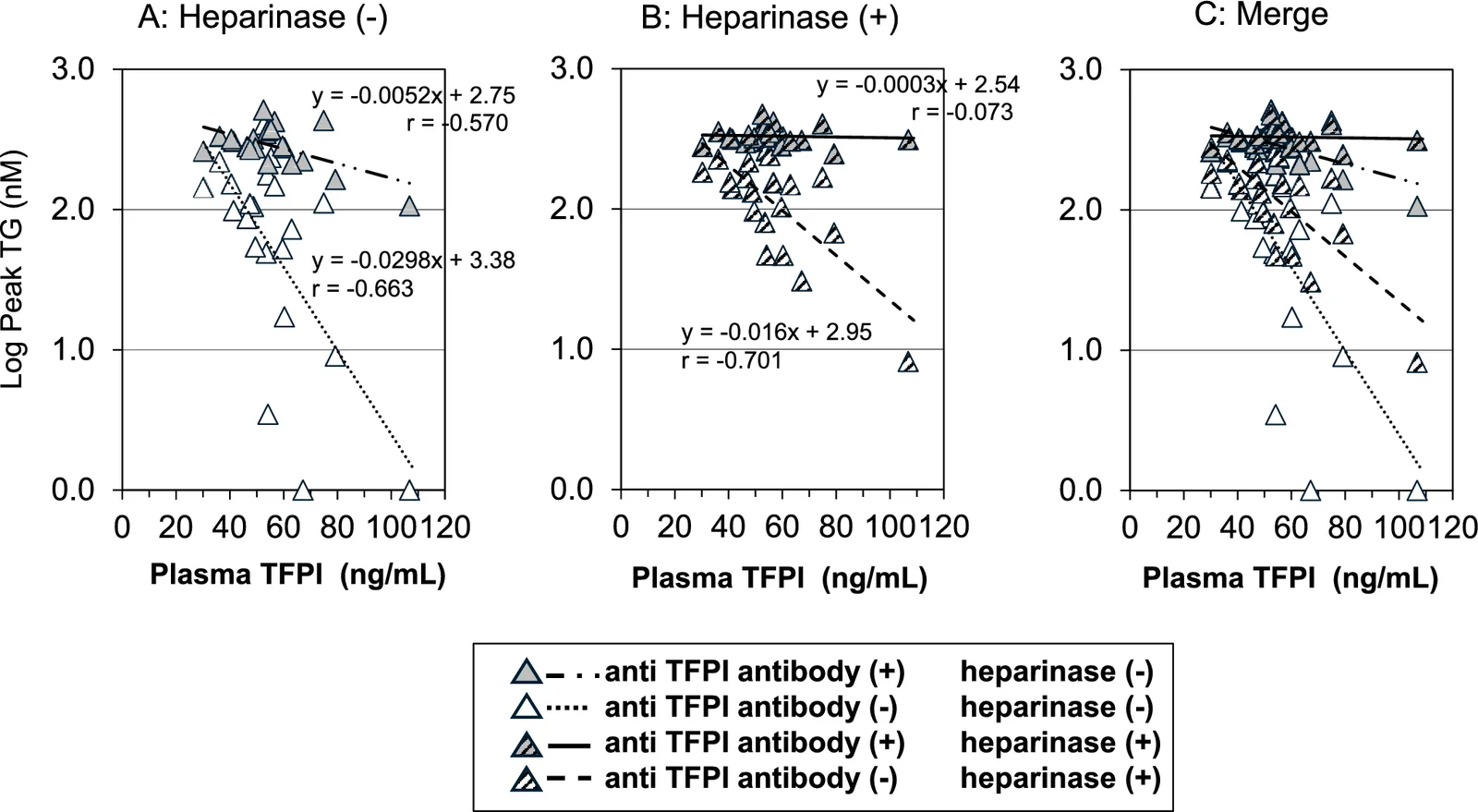
Negative correlation between peak thrombin generation (TG) value and plasma tissue factor pathway inhibitor (TFPI) concentration at the end of surgery. The correlation between peak TG value and plasma TFPI concentration without heparinase (**A**) and with heparinase (**B**). A merge of panels A and B (**C**) shows that peak TG was restored by anti-TFPI antibody treatment with or without heparinase treatment; analysis of covariance showed no significant difference in TFPI-induced inhibition in TG at the end of surgery between these two conditions (P = 0.09). TG, thrombin generation; TFPI, tissue factor pathway inhibitor; POD 1, postoperative day 1

Thus, we hypothesized that the inhibition of TG associated with increased TFPI concentration primarily occurred postoperatively. To further investigate this phenomenon, we examined the effects of TFPI under the condition in which the effect of residual heparin was eliminated by heparinase. Without heparinase treatment, TFPI concentration (C_TFPI_) was negatively correlated with log-transformed peak TG value (log peak TG = C_TFPI_ ×  − 0.0298 + 3.38; r =  − 0.663), and peak TG value decreased in a TFPI-dependent manner, which was attenuated by the addition of anti-TFPI antibody (log peak TG = C_TFPI_ ×  − 0.0052 + 2.75; r =  − 0.570; Fig. 5A).When similar analyses were conducted under heparinase treatment, TFPI concentration still correlated negatively with log-transformed peak TG value (log peak TG = C_TFPI_ ×  − 0.016 + 2.95; r =  − 0.701), and the addition of anti-TFPI antibody abolished the TFPI-dependent reduction in peak TG value (log peak TG = C_TFPI_ ×  − 0.0003 + 2.54; r =  − 0.073; Fig. 5B). Analysis of covariance showed that the effects of TFPI-neutralizing antibody (*P* < 0.001) and heparinase (*P* = 0.01) were significant; however, the interaction between the two factors was not significant (*P* = 0.09; Fig. 5C).

These results indicate that while a TFPI concentration-dependent reduction in peak TG value was observed after surgery, the contribution of residual heparin was not significant. This demonstrates that TFPI suppresses TG at the end of surgery in a concentration-dependent manner.

### Impact of TFPI-induced reduction in peak TG in postoperative bleeding

**Fig. 6:**
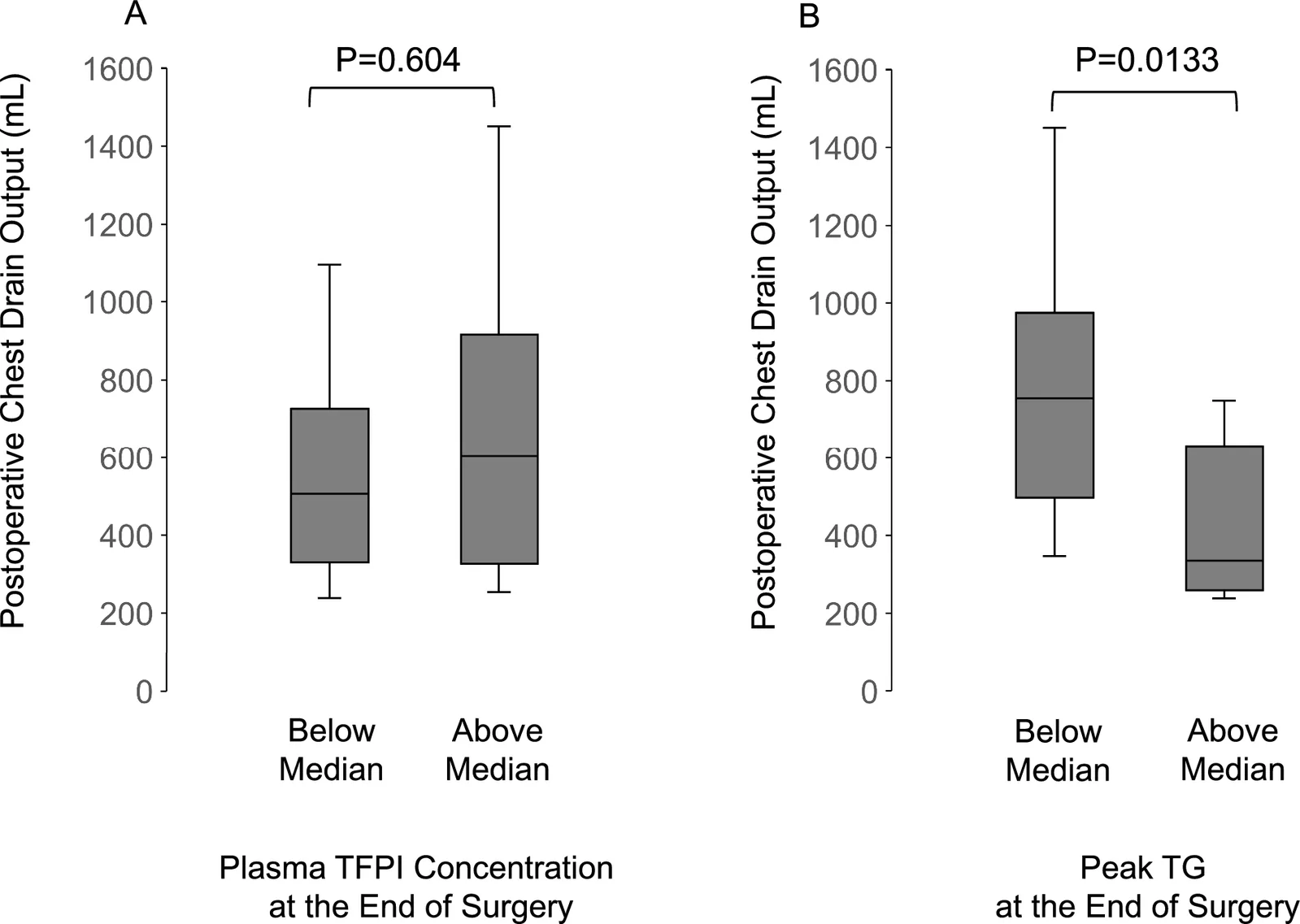
Relationship of 24-h postoperative chest drain output with plasma TFPI concentration and peak TG. Excluding two patients who required re-thoracotomy for hemostasis, 19 patients were analyzed. **A** Chest drain output did not significantly differ between patients with plasma TFPI concentrations below the median (54.0 ng/mL) and those at or above the median (*P* = 0.604). **B** When patients were dichotomized based on the median peak TG value (86.4 nM), chest drain output was significantly higher in the group with low peak TG (*P* = 0.0133). TG, thrombin generation; TFPI, tissue factor pathway inhibitor

The chest drain output 24 h after surgery was 530 [348–834] mL, and 2 of the 21 patients required surgical re-exploration for hemostasis. In both patients, the cause of postoperative bleeding was inadequate surgical hemostasis. The remaining 19 were dichotomized based on median TFPI concentration at the end of surgery (54 ng/mL) and compared in terms of chest drain output at 24 h, which did not significantly differ between the groups (Fig. 6A). In contrast, when patients were dichotomized based on median peak TG value at the end of surgery (86.4 nM), chest drain output at 24 h was significantly higher in the group with peak TG ≤ 86.4 nM (Fig. 6B).

Supplemental Figure S1 shows the results of the correlation analysis between postoperative bleeding and parameters including TFPI, peak TG, fibrinogen concentration, and platelet count. TFPI and peak TG demonstrated moderate correlations, with correlation coefficients of 0.44 and − 0.44, respectively, explaining approximately 19% of the variability in drainage volume. In contrast, the correlation coefficient for fibrinogen concentration was 0.013, indicating a negligible correlation. The coefficient for platelet count was − 0.23, indicating a weak correlation.

## Discussion

This study demonstrated a significant decrease in peak TG value at the end of cardiac surgery using CPB; a major contributing factor was a significant increase in TFPI concentration. Postoperative plasma TFPI concentration was significantly increased from baseline and showed a negative correlation with peak TG value. In addition, this reduction in peak TG was abolished by neutralizing TFPI with antibody.

### Postoperative increase in plasma TFPI concentration following cardiac surgery

Plasma TFPI concentrations at the end of surgery were significantly higher compared with baseline. This finding is in line with previous reports showing that TFPI concentration increases in plasma because of the effect of heparin and does not immediately return to preoperative levels even after neutralization with protamine [8]. Our results therefore do not contradict these earlier observations.

Regarding the degree of elevation in TFPI concentration, the limited number of participants in this study precludes a detailed analysis of contributing factors; thus, further investigation with a larger cohort is warranted. It is also possible that inadequate neutralization of heparin may delay the decline in TFPI concentration, representing one potential mechanism underlying the observed postoperative changes.

### Relationship between anti-factor Xa activity in whole blood and plasma TFPI concentration

The coagulation time measured in whole blood samples after the addition of Russell’s viper venom was prolonged in association with TFPI concentration. We employed a system developed to assess the anticoagulation effects of factor Xa inhibitors using a dielectric blood coagulometer. This device determines coagulation time by monitoring changes in dielectric permittivity under a 1-MHz alternating current, which occurs in association with fibrin formation. The method used in this study employed Russell’s viper venom, a factor X activator, enabling assessment of anti-Xa factor activity via coagulation time measurements. In previous studies, we have demonstrated the usefulness of this methodology in evaluating reduction in TG and determining drug efficacy in patients taking factor Xa inhibitors [7]. Given that TFPI’s anticoagulant effect includes inhibition of factor X, we also consider this system to be useful in evaluation of anti-factor Xa activity in whole blood samples. Our findings indicate that prolongation of coagulation time measured by this system reflected the existence of an antidote to factor Xa, showing strong dependence on plasma TFPI concentration at the end of surgery.

### Contribution of TFPI to reduction of TG in PPP

TG was significantly lower at the end of surgery than at baseline, which has also been demonstrated in several previous studies [2, 3, 8]. Our hypothesis was that TFPI contributes to this reduction. TFPI is a serine protease inhibitor with a single-chain structure containing three Kunitz inhibitory domains (K1, K2, K3) that binds with factors VII and X, inhibiting the early stages of coagulation.

We found no apparent TFPI concentration-dependent reduction in peak TG value in preoperative samples with TFPI concentration ≤ 33 ng/mL, whereas a significant negative correlation between peak TG value and TFPI concentration was observed at the end of surgery. Moreover, the reduction in TG capacity was restored by adding anti-TFPI antibody. Although residual heparin administered during CPB and redistribution from tissues may have also contributed to reduction in TG, the significant negative correlation between TFPI concentration and peak TG value remained significant even after heparinase treatment, which eliminated the effect of residual heparin. In addition, TG was restored by neutralizing TFPI. These results indicate that TFPI contributes to the decrease in peak TG seen after patients have been on CPB. Furthermore, the observation that peak TG returned to the same level in all samples after the effects of heparin and TFPI were neutralized by specific antibodies indicates that depletion or dilution of coagulation factors was unlikely to have been a major factor to explain postoperative reduction in TG in this study setting.

Although physiological peak TG values have not been clearly defined in previous reports, based on control samples and samples spiked with direct oral anticoagulants in previous studies in our laboratory, we believe that the physiological peak TG level is ≥ 100 nM in healthy individuals. In the current study, most of the samples with peak TG < 100 nM had a TFPI concentration > 40 ng/mL. This suggests that concentrations > 40 ng/mL can result in suppression of coagulation activity. Our results from the baseline samples are consistent with those in previous studies reporting the normal concentration range of TFPI [9, 10].

### Impact of changes in TFPI concentration and TG on postoperative bleeding

No correlation was observed between plasma TFPI concentration at the end of surgery and postoperative bleeding. However, when the patients were dichotomized by the median peak TG value, the low TG group showed a tendency for increased bleeding. TFPI is an endogenous anticoagulant that suppresses TG in a concentration-dependent manner, but baseline TG varies among individuals. These results suggest that there exists a threshold level of peak TG required for effective hemostasis, and that whether peak TG falls below this threshold because of the effect of TFPI was more important than the absolute concentration of TFPI itself.

Several previous studies have also demonstrated that postoperative TG influences postoperative bleeding [3, 8]. Bosch et al. pointed out that baseline TG correlates with postoperative bleeding [11]. Accordingly, our findings suggest that in patients with lower baseline TG, TFPI-mediated inhibition may reduce TG below the critical cutoff, thereby increasing the risk of postoperative bleeding.

To address TFPI-induced coagulation impairment, administration of blood products containing factors VII and X may be considered as clinically effective interventions. Our study showed that TG increased after anti-TFPI antibody was added to blood samples in vitro, suggesting that anti-TFPI antibody may have a hemostatic effect. However, the clinical indication for anti-TFPI antibody at present is limited only to hemophilia, and antibody neutralization of TFPI may increase TG beyond physiological levels, posing a risk of hypercoagulability. A phase III study of concizumab, a monoclonal antibody to TFPI initially developed as a treatment for hemophilia, reported thrombotic complications in participants receiving high doses [12]. Instead, TFPI-induced coagulation impairment and reduction in TG can be antagonized by fresh frozen plasma and prothrombin complex concentrate [13].

### Study limitations

This study was exploratory in nature, and no formal a priori sample size calculation was performed. In this context, owing to the limited sample size, it may have been underpowered to detect weak correlations, and the findings should be interpreted with caution.

Furthermore, the surgical procedures performed on these 21 patients were diverse, and multiple factors including cardiopulmonary bypass time may have acted as confounding variables. To address this, methods such as multivariate analysis and subgroup analysis are required, which in turn necessitate recruiting a larger number of subjects. For a more generalizable investigation, it will be necessary to conduct large-scale multicenter studies.

Finally, supplementation of anti-TFPI antibody was conducted ex vivo*,* which may not fully replicate the in vivo effects. In this context, future studies should examine the effectiveness of clinical interventions aimed at reversing the suppression caused by TFPI, such as by neutralization of TFPI or supplementation of coagulation factors suppressed by TFPI.

## Conclusions

Cardiac surgery using CPB is associated with an increase in plasma TFPI concentration at the end of the surgery. This elevation induces a decrease in TG at the end of surgery, which can be a therapeutic target to establish stable hemostasis after cardiac surgery.

## Supplementary Information

Below is the link to the electronic supplementary material.

Supplementary file1 (PPTX 63 KB)

## Data Availability

The datasets used and/or analyzed during the current study are available from the corresponding author on reasonable request.

## Declarations

### Submission declaration

Partial results of this study were presented at the 29th Annual Meeting of the Japanese Society of Cardiovascular Anesthesiologists as an abstract. The contents of this manuscript have not been published elsewhere.
